# Real‐time ancillary diagnostics for intraoperative assessment of intestinal viability in horses–looking for answers across species

**DOI:** 10.1111/vsu.14248

**Published:** 2025-03-20

**Authors:** Nicole Verhaar, Florian Geburek

**Affiliations:** ^1^ Clinic for Horses University of Veterinary Medicine Hannover Hannover Germany

## Abstract

Clinical intestinal viability assessment is associated with significant limitations, and there is an undisputable need for ancillary diagnostics during colic surgery. Human and companion animal surgeons struggle with similar intraoperative issues, yet there is little exchange between specialists. Therefore, this narrative review aimed to create an overview of real‐time ancillary diagnostics with the potential for intraoperative intestinal viability assessment in horses. Most real‐time ancillary diagnostics can be classified as either tissue perfusion or oxygenation assessments. Intestinal perfusion may be quantified using dark field microscopy, laser Doppler flowmetry, or fluorescence angiography (FA). In particular, indocyanine green FA has gained popularity in human medicine and is increasingly employed to predict intestinal injury. Intestinal oxygen saturation can be measured by pulse oximetry or mixed tissue oximetry. The latter can be conducted using visible light or near‐infrared spectrophotometry, and these measurements correlate with clinical outcomes in various species. Other real‐time diagnostics include thermography and techniques currently under development, such as laser speckle flowgraphy or photoacoustic imaging. The modalities discussed are minimally invasive and may be used for intraoperative assessments of the intestine. However, limitations include the occurrence of artifacts and the subjective nature of some modalities. Techniques such as indocyanine green FA and tissue oximetry are already available in veterinary practice and have the potential for use during colic surgery. However, blinded clinical trials are lacking in all species, and more research is needed to determine the accuracy and cutoff values in equine‐specific intestinal lesions.

**Abbreviations**
AMI: acute mesenteric ischemia
FA: fluorescence angiography
Hb: hemoglobin
ICG: indocyanine green
LDF: laser Doppler flowmetry
NIR: near‐infrared
NIRS: near‐infrared spectrophotometry
POX: pulse oximetry
PPG: photoplethysmography
StO_2_
: tissue oxygen saturation
ViS: visible light spectrophotometry

## 1 INTRODUCTION

Intestinal viability assessment during colic surgery is currently based on clinical judgment.^1^, ^2^ For this evaluation, serosal or mucosal color, intramural hemorrhage, strictures, wall thickness, and intestinal contractions can be assessed.^1^, ^2^, ^3^ Furthermore, the pulse in the mesenteric arteries and hemorrhage at an enterotomy site can be used as measures for local macroperfusion.^1^, ^2^, ^3^ However, clinical assessment was shown to be unreliable for the prediction of injury in experimental models of small intestinal ischemia.^4^ Furthermore, clinical studies have reported injury progression in small and large intestinal segments that looked viable, as well as segments deemed unviable that survived.^5^, ^6^, ^7^ A semiquantitative score has been published to enable a more outcome‐oriented clinical assessment of the small intestine.^3^ Nonetheless, this cannot predict the progression of injury, and the applicability of this score in the ileum and colon remains unclear.

**FIGURE 1:**
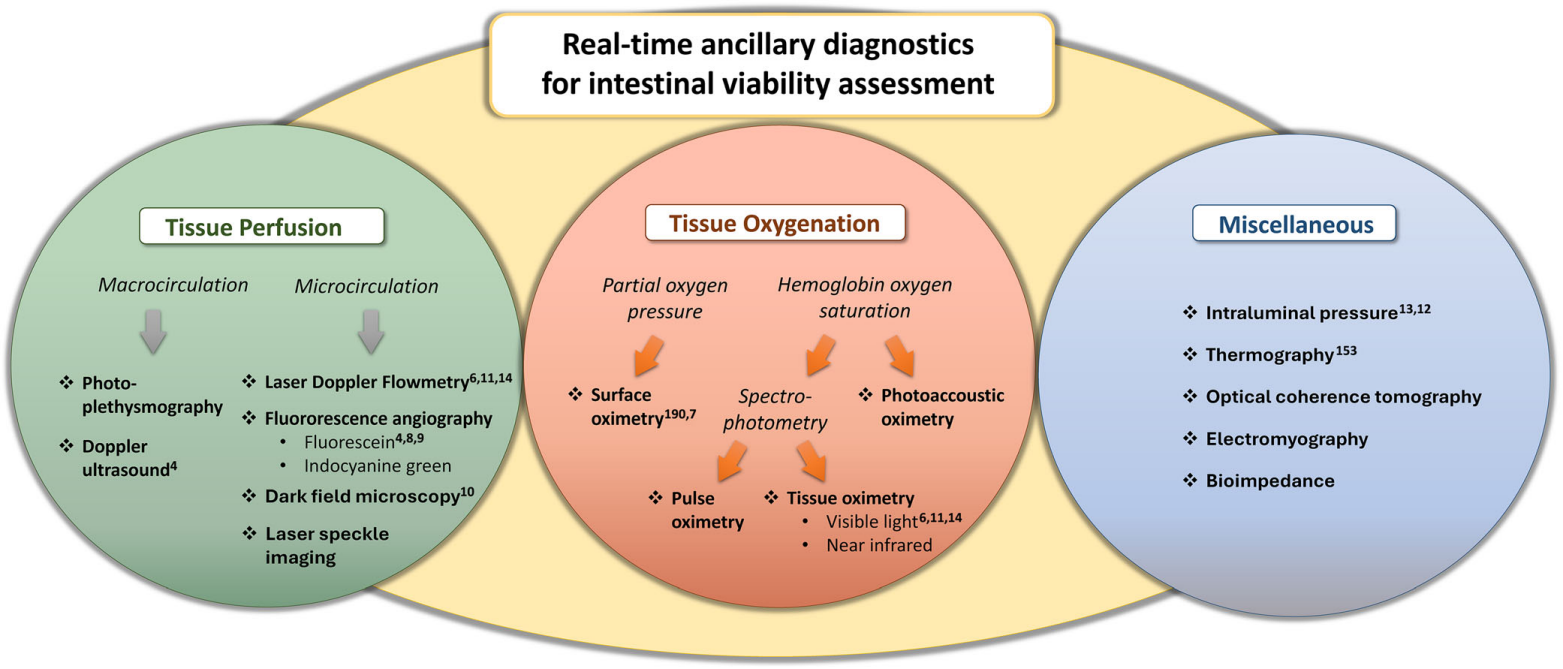
Diagram depicting the possible ancillary methods for intraoperative viability assessment of ischemic intestine. Sources that have reported the use of the modality in horses for intestinal viability assessment in experimental or naturally occurring intestinal ischemia are referenced as superscript numbers. Freeman et al.,^4^ Verhaar et al.,^6^ Verhaar et al.,^11^ Verhaar et al.,^14^ Sullins et al.,^8^ Brusie et al.,^9^ Hurcombe et al.,^10^ Snyder et al.,^190^ Snyder et al.,^7^ Moore et al.,^13^ Mathis et al.,^12^ Purohit et al.^153^

Despite the questionable accuracy of clinical viability assessment,^4^ reliable ancillary diagnostics are lacking. A handful of studies have investigated different modalities for application in the ischemic equine intestine (Figure 1).^4^, ^6^, ^7^, ^8^, ^9^, ^10^, ^11^, ^12^, ^13^, ^14^ However, most studies date back to the previous century, and experimentally tested diagnostics have not found their way into clinical practice. The limitations of clinical viability assessment are also apparent in other species, with the accuracy for predicting irreversible intestinal injury ranging between 36% and 73% in experimental animal models.^15^, ^16^, ^17^, ^18^, ^19^, ^20^, ^21^ In human medicine, intestinal injury has also been over‐ or underestimated, leading either to unnecessary intestinal resection or to an increased risk of postoperative complications.^22^, ^23^, ^24^, ^25^, ^26^ Therefore, one can conclude that human and veterinary surgeons struggle with comparable intraoperative issues.

When comparing intestinal viability assessment between species, interspecies differences in pathogenesis and indications for intraoperative viability assessment should be considered. In dogs, foreign body obstruction with pressure necrosis of the intestinal wall is an important indication for intestinal viability assessment.^27^ In human medicine, colorectal resection and anastomosis for cancer pose a significant risk for poor vascularization associated with ischemia and concurrent anastomotic leakage.^28^ These ischemic lesions do not resemble the hemorrhagic strangulating obstructions typically seen in horses.^2^ However, intestinal diseases such as volvulus, hernias, and intussusceptions occur in all species.^29^, ^30^, ^31^, ^32^, ^33^, ^34^, ^35^, ^36^, ^37^, ^38^, ^39^, ^40^ Despite this overlap in intestinal disease and the concurrent issue of viability assessment, there is little exchange of methods between respective specialists. Therefore, this narrative review aimed to create an overview of the real‐time ancillary diagnostics for intestinal viability assessment, including the potential and availability for use during equine colic surgery.

## 2 MATERIALS AND METHODS

The online databases Pubmed, CAB Abstracts, and Google Scholar were searched using combinations of “intestine,” “bowel,” “viability,” and “assessment.” Based on this search, a list of potential diagnostic modalities was compiled. Subsequently, further searches were conducted in the aforementioned databases using all individual modalities along with their synonyms, in combination with “equine,” “horse,” “intestine,” “bowel,” “strangulation,” or “obstruction.” During the preparation of the manuscript, additional searches were undertaken to gather further background information on the respective techniques. There was no restriction on the date of publication; however, papers written in a language other than English or German were excluded.

The titles and abstracts were evaluated for eligibility, and papers were included if they met the following criteria:

- Prospective or retrospective studies on the intraoperative use of a real‐time measuring technique at some location in the gastrointestinal tract, with the goal of viability assessment in either experimental or naturally occurring ischemia of any species.
- Studies were also included if the technique was applied in healthy, non‐ischemic intestine in the horse.

The following papers were excluded:

- Papers discussing modalities, such as the retrieval of microspheres, that are solely utilized for experimental purposes without any perspective for clinical use.
- Papers reporting histology as a single diagnostic modality.
- Retrospective single case reports in human medicine in the presence of >5 larger case series of the same diagnostic modality.

## 3 REAL‐TIME ANCILLARY DIAGNOSTICS WITH CLINICAL AVAILABILITY

Most techniques for assessing ancillary intraoperative intestinal viability quantify either tissue perfusion or tissue oxygenation (Figure 1). In addition, techniques that quantify tissue injury, metabolism, and motility were included in the miscellaneous category. The included modalities were restricted to those that are commercially available for clinical practice.

### 3.1 Perfusion

**FIGURE 2:**
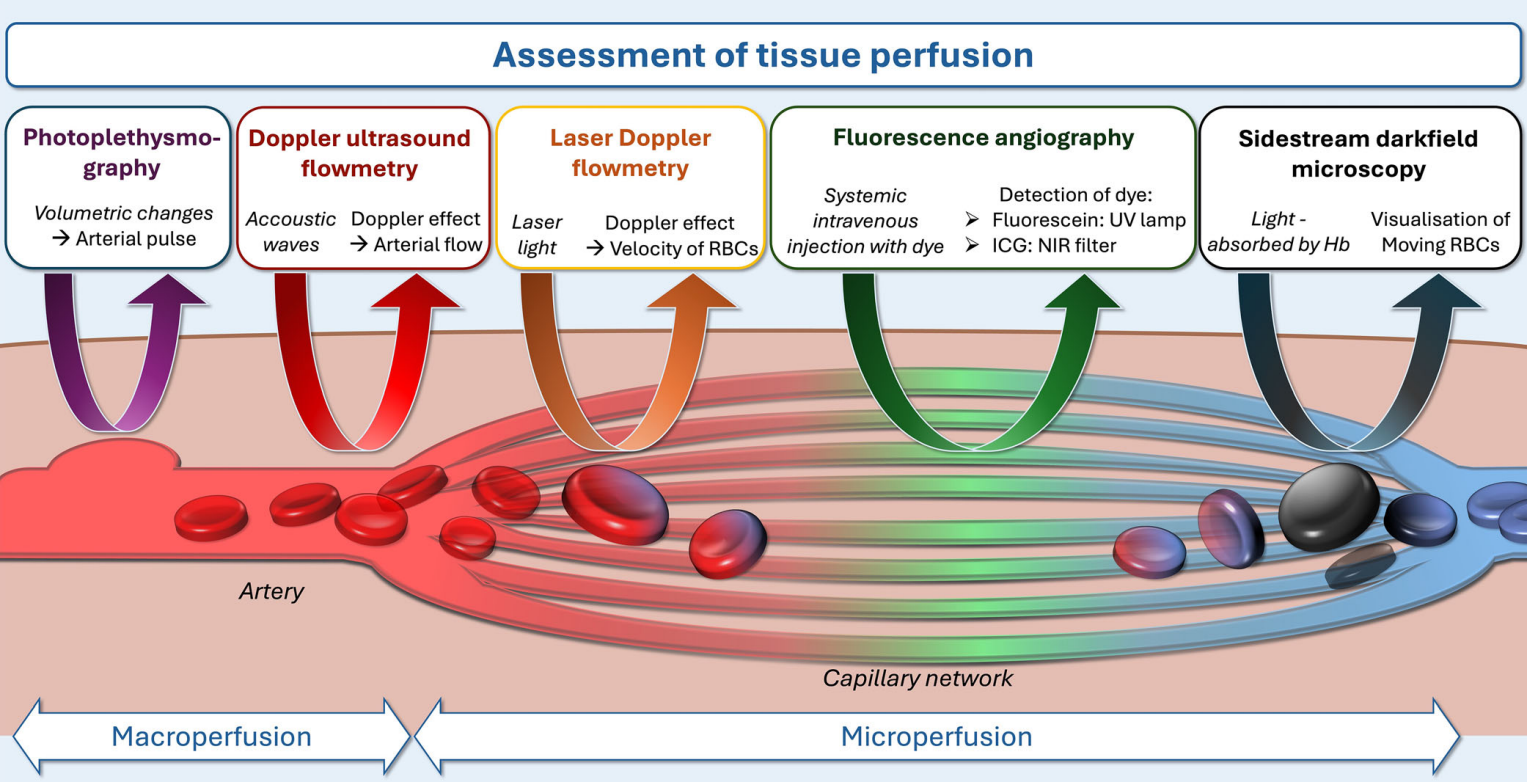
Schematic illustration depicting common techniques to assess tissue perfusion in the intestine. Hb, hemoglobin; ICG, indocyanine green; NIR, near infrared; RBCs, red blood cells; UV, ultraviolet.

Perfusion can be used as a measure of intestinal viability, and microvascular patency is associated with outcome after ischemic injury.^41^ Because visual and palpatory assessment of perfusion is not considered reliable, minimally invasive diagnostic modalities have been developed to quantify tissue blood flow. Important characteristics of these modalities are the ability to obtain qualitative or quantitative measurements and whether they reflect either macro‐ or microperfusion (Figure 2). A quantitative assessment of microperfusion would be preferable as an objective and representative measure of tissue damage.

#### 3.1.1 Photoplethysmography

Photoplethysmography (PPG) detects volumetric changes of blood in the tissue, and it can therefore, be used to measure peripheral macroperfusion.^42^ This technique is available in clinical practice because it is applied with pulse oximetry (POX) for anesthetic monitoring.^43^, ^44^ This modality has also been used without oximetry to assess intestinal perfusion in an experimental model of canine ischemia.^45^, ^46^ Limitations are the semiquantitative nature of the measurement and the detection of macroperfusion instead of microperfusion. Furthermore, blood flow under a certain threshold cannot be detected, thereby limiting the sensitivity of this instrument.^46^ Because of these limitations, PPG is less suitable for intestinal viability assessment despite its widespread availability.

#### 3.1.2 Doppler flowmetry

Doppler ultrasound flowmetry was the first to apply the Doppler principle to assess intestinal viability. This technique has been implemented with varying results in experimental ischemia models in horses, dogs, rabbits, baboons, and human patients.^4^, ^19^, ^21^, ^22^, ^41^, ^46^, ^47^, ^48^, ^49^, ^50^, ^51^, ^52^, ^53^, ^54^, ^55^, ^56^, ^57^, ^58^, ^59^, ^60^, ^61^, ^62^, ^63^, ^64^ It was less reliable than other ancillary diagnostics,^45^, ^61^, ^62^, ^63^, ^64^ and only yielded non‐quantitative measurements of local macroperfusion.^22^, ^46^ Because of these limitations, the device has not found its way into clinical practice.

In contrast to Doppler ultrasound flowmetry, laser Doppler flowmetry (LDF) is a quantitative measurement of microperfusion, also referred to as laser Doppler velocimetry or fluxmetry. This modality is not routinely used in veterinary or human clinical practice, but devices are commercially available. This technique determines the velocity of the erythrocytes by detecting the Doppler shift induced by laser light on the moving erythrocytes. This is then used to quantify tissue blood flow.^65^ The measurement can be obtained within seconds through direct contact with a probe on the serosal side of the intestine. The main limitation of this technique is the risk of significant motion artifacts that may occur due to intestinal motility or mechanical ventilation of the horse.^66^, ^67^ Furthermore, severe intramural hemorrhage could interfere with the light reflection, resulting in a limited penetration depth or the inability to obtain measurements in such cases.^6^ One should also be aware of the small field of view, necessitating multiple measurements to obtain information on the microperfusion in a larger area. Routine LDF has been adapted to measure larger tissue sections by scanning LDF.^68^ However, this is not commercially available.

Evaluating the available evidence in horses, a device combining LDF with tissue oximetry has been used in naturally occurring intestinal strangulations during colic surgery.^6^, ^11^, ^14^ One study, including 40 horses with small intestinal lesions, found lower tissue blood flow values in the affected segments compared to measurements in healthy anesthetized horses.^6^, ^69^ Furthermore, segments with more severe histological injury were associated with lower blood flow measurements.^6^ Although cutoff values could not be determined due to the low sample size, tissue blood flow values <5% of previously determined reference values were indicative of intestinal necrosis.^6^, ^69^ Another clinical study, including 18 horses with large colon volvulus and descending colon strangulations, found lower colonic blood flow in non‐survivors compared to survivors.^14^

In studies evaluating LDF in other species, a clinical trial in dogs with gastric dilatation and volvulus found that gastric tissue blood flow was lower in cases that required partial gastrectomy compared to those that did not necessitate resection.^36^ Furthermore, LDF correlated well with histology, anastomotic leakage, and submucosal blood flow quantified with hydrogen gas clearance in experimental studies in dogs, pigs, cats, and rats.^70^, ^71^, ^72^, ^73^, ^74^, ^75^ A comparison of LDF to other modalities showed that LDF was more reliable than Doppler ultrasound flowmetry and POX.^18^, ^62^, ^63^ Rabbit and canine studies comparing LDF to fluorescence angiography (FA) provided conflicting results, with both lower and higher accuracies for LDF reported in these studies.^18^, ^62^

Laser Doppler flowmetry has also been applied in human patients with acute mesenteric ischemia (AMI) and intestinal strangulations. The modality was more accurate than clinical assessment in both small and large intestines, and it enabled the resection of shorter intestinal segments.^25^, ^76^ Furthermore, LDF could identify severe ischemic injury in the jejunum that was underestimated by clinical assessment.^77^, ^78^

In summary, LDF has been successfully applied in horses with naturally occurring colic to identify intestinal ischemia, but more research is needed to determine its accuracy. Artifacts may be overcome by measuring at different locations and for more extended periods.^6^ However, due to its susceptibility to motion artifacts, this technique requires handling by a trained operator, and the results need to be interpreted in the context of the intraoperative findings.

#### 3.1.3 Fluorescence angiography

Fluorescence angiography assesses intestinal microperfusion by evaluating the fluorescence pattern in the intestine following intravenous injection of dye agents. Older studies have used fluorescein as dye, assessing perfusion by the illumination of the intestine with ultraviolet light.^4^ In the past decade, indocyanine green (ICG) dye has gained popularity in human medicine.^28^, ^79^, ^80^, ^81^ Indocyanine green is visualized using a near‐infrared (NIR) imaging device or filter, and it can easily be applied in an endoscopic setting because no special light source is needed. Furthermore, vessels located deeper within the tissue can be visualized with this dye.^82^ The fluorescence pattern can be evaluated within minutes following intravenous dye injection, either by direct visual qualitative evaluation or by quantitative fluorimetry.^83^, ^84^ Vessels that are not perfused cannot be visualized, so it is impossible to determine the proportion of perfused arterioles and capillaries contributing to the fluorescence.^85^ With visual analysis, there is a lack of objectivity and poor agreement between operators, even with standardized protocols.^22^, ^86^, ^87^ This can be improved using quantitative fluorimetry to monitor dye elimination and uptake or by implementing artificial intelligence for pixel analysis.^88^, ^89^ Another limitation of this technique is the persistence of dye in the tissue. Therefore, the measurement cannot be repeated within a short timeframe. Quantitative fluorimetry may solve this issue by subtracting the remaining fluorescence of previous injections from new post‐injection values.^88^ It has been hypothesized that FA is less applicable in thicker tissue, such as the stomach wall,^36^ yet primary sources are lacking. No adverse reactions to dye application have been described.

In horses, only fluorescein dye has been used for intestinal viability assessment. One equine experimental small intestinal study found that FA had a worse overall accuracy than Doppler ultrasound flowmetry.^4^ Another small intestinal study reported both patchy and normal fluorescence patterns in ischemic segments. More importantly, these segments were not normal at second‐look laparotomy 1 month after the first surgery, thereby questioning the sensitivity of this technique to detect irreversible changes.^8^ Fluorescein FA could differentiate small intestinal hemorrhagic strangulating obstructions from uninjured control segments and ischemic strangulating obstructions. However, FA could not distinguish between ischemic strangulating obstructions and uninjured control segments.^9^ Consequently, FA appeared to be less reliable in ischemic strangulating obstructions. There are no reports on using FA in naturally occurring ischemia in horses.

Evaluating the evidence in other animals, there is only one report of the clinical use of FA in the canine intestine. These authors reported the application of ICG for the resection of intestinal neoplasia but not for viability assessment.^90^ In experimental ischemia in rats, pigs, and dogs, fluorescein FA was more reliable than clinical assessment, and FA could differentiate between ischemic and viable bowel segments in both open surgery and laparoscopic settings.^41^, ^85^, ^91^, ^92^ However, it should be noted that two studies identified hyperfluorescent patterns that incorrectly suggested viability in intestinal segments that would progress to necrosis.^83^, ^88^ According to a study using ICG FA in rats and pigs with experimental small intestinal ischemia, a filling defect in the fluorescence pattern was 85% accurate in predicting histological injury grade.^84^

In human medicine, fluorescein FA has successfully been used for intestinal viability assessment in AMI and strangulating lesions, as well as for the transplantation of a free jejunal graft for esophageal resection.^22^, ^26^, ^58^, ^93^ In two of these studies, FA was more accurate than Doppler ultrasound.^22^, ^58^ Furthermore, FA could predict intestinal perforation in patients with intramural hemorrhage following abdominal trauma.^94^ Nonetheless, all studies using fluorescein as a dye date back to the previous century, and all newer reports have focused on ICG FA. Looking at the available evidence for the use of ICG FA in patients with strangulating lesions or AMI, only one prospective trial was published. In 38 patients with strangulating lesions, a laparotomy with clinical assessment only was compared to a laparoscopic procedure with ICG FA. The authors reported a higher complication rate in the laparotomy group, but the allocation of the patients was not randomized, thereby limiting the value of this trial.^95^ Furthermore, many authors have reported the use of ICG FA during laparotomy or laparoscopy without comparing it to other techniques.^87^, ^96^, ^97^, ^98^, ^99^, ^100^, ^101^, ^102^ The main result of these case series was that ICG FA was a useful intraoperative tool to determine the need for and/or the location of intestinal resection.^87^, ^96^, ^97^, ^98^, ^99^, ^100^, ^101^, ^102^ No adverse effects were reported, and the additional surgery time was considered minor. The only negative finding was that ICG FA underestimated ischemic injury in 3/91 and 2/52 patients in two larger case series.^101^, ^102^ Despite these positive reports, a recent survey among emergency surgeons found that ICG FA is still not established as a routine technique in an emergency setting.^103^ This is most likely the result of limited training, the availability of NIR imaging during emergency surgeries, and a lack of standardized protocols for this procedure. The main indication to use ICG FA in human medicine is to evaluate the viability and determine the location of resection margins in colorectal surgery. Although one randomized controlled multicenter clinical trial failed to identify a difference in anastomotic leakage rate,^104^ meta‐analyses based on retrospective studies have generally concluded that the use of ICG FA reduces the occurrence of anastomotic leakage.^28^, ^79^, ^80^, ^81^

In summary, this technique may be suitable as an intraoperative ancillary diagnostic during colic surgery despite the identified limitations. The clearance of ICG has been investigated in horses,^105^, ^106^ and ICG has been used for ocular FA and experimental endoscopic laser surgery of upper airway tissue.^107^, ^108^ Modern endoscopy units in equine practice can be equipped with NIR filters, and ICG dye is readily available. Based on one early study using fluorescein as a dye, FA may be more useful in hemorrhagic strangulating obstructions.^9^ Still, more research is needed to determine the applicability of (ICG)FA in naturally occurring intestinal diseases. The subjective nature of visual assessment remains a significant limitation. Quantitative methods may improve this, but these are currently unavailable in clinical practice.

#### 3.1.4 Sidestream darkfield microscopy

Sidestream darkfield microscopy is based on the principle of orthogonal polarization spectral imaging. With this technique, the tissue is illuminated with a wavelength absorbed by hemoglobin, making red blood cells appear dark. This enables visualization of the red blood cells flowing through the tissue.^109^ These microperfusion measurements are performed with a videoscope handpiece coupled to a monitor for real‐time evaluation. Devices are commercially available but not routinely used in clinical practice. The main limitation of this technique is the superficial nature of the measurements, which only reflect serosal microperfusion. Furthermore, bowel peristalsis and pressure of the device on the tissue surface can lead to significant artifacts.^110^

In horses, darkfield microscopy has been applied during colic surgery. Perfusion patterns were compared between 9 control horses and 23 horses with different large intestinal diseases, including simple obstruction, non‐strangulating colon displacement, and large colon volvulus.^10^ Although differences were found between the cases with simple obstructions and strangulating lesions, dark field microscopy could not differentiate between strangulating and non‐strangulating colon displacements.^10^

Looking at the evidence in other species, dark field microscopy failed to detect a difference between viable and nonviable intestinal segments in dogs with intestinal foreign body obstructions.^111^ In contrast, one human study found lower perfusion indices in patients with colorectal anastomotic leakage compared to patients without anastomotic leakage.^112^, ^113^

In conclusion, it is technically feasible to apply darkfield microscopy during colic surgery, but based on the current evidence, the sensitivity and diagnostic value of this modality may be limited.

### 3.2 Oxygenation

**FIGURE 3:**
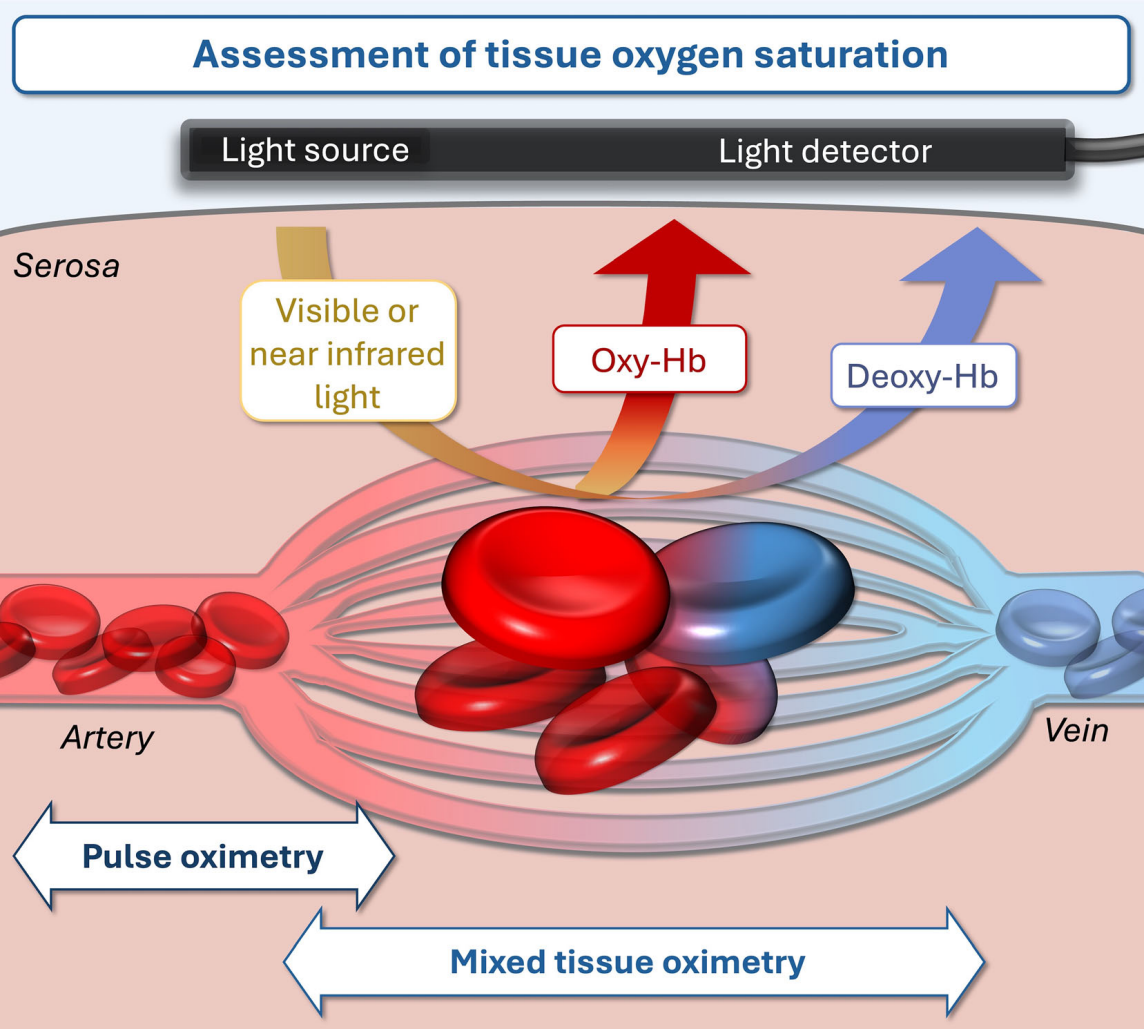
Schematic illustration depicting techniques to assess oxygen saturation of the intestine. Hb, hemoglobin.

The oxygenation of intestinal tissue reflects the quality of tissue perfusion and may be used as a measure for tissue injury and the ability to restore blood flow.^114^ Tissue oxygen saturation is determined by the ratio of oxygenated and deoxygenated hemoglobin (Figure 3). This can be quantified by spectral measurements referred to as spectrophotometry or spectroscopy. The spectral properties of horse hemoglobin are nearly identical to those of other species, including humans, thereby facilitating interspecies comparison and the use of devices developed for human application.^115^, ^116^ Many oximetry devices are readily available in veterinary practice because of their use in anesthetic monitoring. Measurements are performed in real time through direct contact with the intestinal tissue. To ensure sterility, transparent ultrasound sleeves have been used around the sensor without any reported effects on the measurements,^6^, ^117^ but the risk of light attenuation should be considered. Oximetry is less susceptible to motion artifacts than flow measurements, but severe intramural hemorrhage, local pigmentation, and ambient light can interfere with spectrophotometric measurements.^43^, ^44^, ^69^, ^118^

#### 3.2.1 Pulse oximetry

Pulse oximetry assesses arterial oxygen saturation in the tissue by combining the principle of spectrophotometry with PPG. As stated previously, PPG determines the peak of the arterial pulse, enabling the calculation of arterial saturation in the tissue at this time.^43^, ^44^ In addition to the limitations mentioned above for spectrophotometric measurements, POX may fail to obtain readings in tissue with local vasoconstriction or decreased blood flow.^43^, ^44^, ^118^

In horses, POX has only been applied to the small and large intestines of healthy individuals.^116^ It was possible to measure oxygen saturation in the equine intestine, but POX measurements were lower than calculated arterial measurements.

In other species, POX has also been used to assess ischemic intestine. The only available clinical animal study was performed in cows, investigating the use of intestinal POX in cases with abomasal displacements.^119^ Decreasing saturation was associated with increasing intraluminal pressures, but the study did not correlate the measurements with histology or outcome. Several experimental studies in models of canine small intestinal ischemia found that POX correlated well with mucosal injury.^61^, ^120^, ^121^, ^122^, ^123^ One study indicated a cutoff value of 70% to predict anastomotic leakage.^120^ In contrast to the positive results in dogs, experimental studies in rabbit small intestines reported relatively low accuracies of 58%–76% in detecting ischemic intestines.^18^, ^20^, ^124^, ^125^

In human medicine, there have been larger clinical trials applying POX in patients undergoing colorectal cancer surgery. These studies found that patients with <90% oxygen saturation at the resection margins were more likely to develop anastomotic leakage.^126^, ^127^ The application of POX in human strangulating intestinal disease has only been documented in single case reports. These authors found that POX was useful in determining the resection location in a colon volvulus and a strangulated stomach.^128^, ^129^

In summary, despite several positive reports and the widespread availability of POX in clinical practice, it is not generally used for intestinal viability assessment. This technique may be useful in individual cases, but its low accuracy in the presence of decreased blood flow or vasoconstriction would limit its applicability in many clinical cases.

#### 3.2.2 Tissue oximetry

Tissue oximetry differs from POX because it assesses the mixed tissue saturation (StO_2_) independent of the arterial pulsative signal. For this purpose, the spectral properties of total tissue hemoglobin are determined by diffuse reflectance spectrophotometry. These measurements are performed with either near‐infrared light spectrophotometry (NIRS) or with white or visible light spectrophotometry (ViS). Mainly NIRS is commonly used in human practice to monitor muscle and cerebral StO_2_ during anesthesia,^130^ and it has also been used for this purpose in horses.^131^, ^132^, ^133^ Compared to ViS, NIRS can obtain deeper measurements due to its longer wavelength.^134^ Generally, these are larger monitoring systems with sensors connected by cables. However, small handheld devices are now available for intraoperative use in humans.^135^, ^136^

Evaluating the evidence in horses, a device combining ViS and LDF has been applied in naturally occurring small and large intestinal strangulating lesions. Similar to the results of the LDF measurements described in the perfusion section, the affected intestines exhibited lower StO_2_ values compared to reference values.^6^, ^14^, ^69^ In the small intestinal study, no significant difference in StO_2_ could be found between different degrees of histological injury, possibly caused by the low number of horses with mild histological injury. The StO_2_ was also measured in the small intestinal segment orad to the strangulation, and cases with StO_2_ <35% were more likely to suffer from postoperative reflux compared to cases with StO_2_ >69%.^11^ In the large intestinal study, survivors had significantly higher colonic StO_2_ values than non‐survivors. Similar to the LDF perfusion measurements, StO_2_ values <10% of previously determined reference values were indicative of intestinal necrosis.^6^, ^14^ These studies did not calculate the sensitivity and specificity of this modality because the case numbers were too low. It should also be noted that StO_2_ could not be measured in some of the cases with severe intramural hemorrhage.^6^, ^14^ One other equine study investigated the association between StO_2_ and small intestinal wall thickness but found no correlation.^137^ Devices using NIRS technology have not been applied in the equine intestine.

Looking at the available literature in other animals, ViS and NIRS have been applied in experimental ischemia in rats and pigs. Tissue oxygen saturation of severely affected segments did not return to baseline, and StO_2_ aided in predicting anastomotic leakage and the survival of free jejunal grafts.^117^, ^138^, ^139^, ^140^, ^141^, ^142^, ^143^

In human medicine, both ViS and NIRS have been used in patients undergoing colorectal surgery. Anastomoses with increasing StO2 values during surgery would go on to heal uneventfully.^144^ Furthermore, a StO_2_ of <60% was indicative of anastomotic complications.^144^, ^145^, ^146^ Two studies using ICG FA as the gold standard found sensitivities and specificities >90% with a handheld NIRS device, and none of the patients suffered any anastomotic complications.^135^, ^136^

In summary, the evidence suggests that ViS and NIRS may be useful for intraoperative viability assessment, with increasing availability in clinical practice due to their use in anesthetic monitoring. Nonetheless, it should be noted that measurements may not be feasible in all cases with severe intramural hemorrhage. Higher case numbers are necessary to assess the accuracy in different types of strangulating lesions, and cutoff values must be determined before widespread clinical use.

### 3.3 Miscellaneous

#### 3.3.1 Intraluminal pressure

Intraluminal pressure measurements can quantify intestinal distention, and this has been proposed as a possible indicator of intestinal damage in horses with large colon volvulus.^12^ This technique can be performed by introducing a needle into the intestinal lumen, connected by tubing to a manometer to measure intraluminal pressure as a measure for colonic distention in a real‐time fashion. The initial report, including 69 horses with non‐strangulating and strangulating obstructions of the ascending colon, yielded promising results with sensitivity and specificity of around 90% to detect non‐survival.^13^ However, another research group found this technique inaccurate in predicting survival in 57 horses with large colon volvulus.^12^ To the authors' knowledge, this modality has not been applied as a diagnostic parameter for intestinal viability assessment in other species. How intraluminal pressure would relate to intestinal viability in different disease entities and anatomical sections remains questionable. This technique may be applicable in certain large intestinal cases to determine the postoperative prognosis, but it will not be of use as a general measure of intestinal viability.

#### 3.3.2 Thermography

Thermography uses an infrared camera to detect changes in tissue surface temperature, which can be used as an indicator for tissue metabolism and perfusion.^147^ It is a widely available and cost‐effective technique, but some limitations should be considered. The ambient environment influences the measurement, and factors such as angle and distance affect the thermal image.^148^, ^149^ The analysis of the color patterns can be done visually, but quantitative software analysis allows for a more detailed and objective evaluation.^150^

In the horse, this technique is available for orthopedic indications,^151^, ^152^ but it is not used during abdominal surgery. One preliminary experimental equine study reported temperature differences between the mesenteric border of the ischemic and nonischemic bowel, possibly indicating inadequate revascularization.^153^ However, there are no reports on the application of thermography in clinical cases during colic surgery.

Evaluating the evidence in other species, reactive hyperthermia following ischemia was associated with survival in small intestinal experimental ischemia in dogs and rats.^15^, ^154^, ^155^ Furthermore, intestinal surface temperature was evaluated in 49 dogs undergoing surgery for small intestinal foreign body obstruction.^150^ There was a decrease in temperature over the foreign body following the resolution of the obstruction, but the study did not assess the association with histology or outcome. In a porcine model for colon anastomoses, thermography was more reliable than ICG FA, especially when evaluating the return to normal temperature following active cooling of the intestine.^156^ Thermography has also been used for viability assessments in human patients during colorectal surgery. Here, it was successfully used for sequential viability assessments in addition to ICG FA.^157^, ^158^, ^159^

Thermography could be feasible for intraoperative viability assessment due to its availability in clinical practice and noninvasiveness. However, the risk of inconsistent measurements due to differences in environment and operator handling needs to be considered. Furthermore, current evidence may support the use of thermography in ischemic strangulating obstructions, but studies in hemorrhagic strangulating obstructions are lacking.

#### 3.3.3 Electromyography

Electromyography can be used as a measure of intestinal viability by assessing the contractile ability of the intestine in response to electric stimuli. In equine medicine, this technique is currently only used for measurements in skeletal muscle to diagnose myopathies and neuropathies.^160^ Larger veterinary facilities may be equipped with this device, mainly for diagnostic purposes in small animal neurology.^161^, ^162^ There is some evidence for the diagnostic value of electromyography in rabbit and canine ischemia models, as well as in human patients with intestinal ischemia.^21^, ^53^, ^54^, ^163^, ^164^, ^165^, ^166^ However, electromyography cannot be considered a non‐invasive diagnostic because it necessitates puncturing the tissue, with reported complications in musculoskeletal tissue.^167^ An adaptation of this technique using surface electrodes has been described, but to the authors' knowledge, this has not been applied for the purpose of intestinal viability assessment.^168^

#### 3.3.4 Bioimpedance measurements

Comparable to electromyography, bioimpedance applies an electric current to the tissue, but instead of measuring tissue contractility, bioimpedance measures tissue resistance as an indicator of tissue composition.^169^, ^170^ Surface electrodes are used for this purpose; hence, puncturing of the tissue is not necessary. Commercial devices have been used in horses in experimental settings to assess body composition, transcranial impedance, and the state of muscle tissue.^171^, ^172^, ^173^ However, bioimpedance has only been applied in the intestine in a porcine ischemia model and in human intestines ex vivo.^174^, ^175^ In these studies, bioimpedance could differentiate ischemic from normal intestine, and machine learning was shown to increase the reliability of this assessment.^175^ However, it has not been applied in naturally occurring ischemia cases, and its applicability in intestinal viability assessment remains unclear.

## 4 TECHNIQUES IN DEVELOPMENT

In addition to the techniques mentioned in the previous section, new modalities have been developed to meet the need for improved ancillary diagnostics. These techniques have not been applied in the equine intestine and are currently unavailable in clinical practice. However, these modalities may hold promise for future applications and are briefly summarized in the following section.

### 4.1 Laser speckle contrast flowgraphy

Laser speckle contrast flowgraphy measures the movement of red blood cells by analyzing speckle patterns generated by the reflection of laser light.^176^ It relies on a principle similar to LDF, but it is used in a non‐contact fashion, creating a velocity map of the blood flow.^176^ Consequently, it can measure a much greater tissue area than LDF. In a porcine model for colon anastomoses, laser speckle contrast imaging was used to assess local intestinal perfusion in a laparoscopic setting.^177^, ^178^, ^179^, ^180^ Subsequently, the technique was applied in human patients undergoing colorectal anastomosis.^181^, ^182^, ^183^ It could detect differences in perfusion after the ligation of marginal vessels, and inter‐rater reliability was high. However, the use of this device did not reduce the incidence of anastomotic leakage. A limitation of this technique is the semiquantitative and subjective nature of speckle pattern assessment, which is also influenced by the positioning of the imaging device.^176^, ^184^ Artificial intelligence has been used to facilitate a more quantitative analysis and may increase the accuracy.^179^

### 4.2 Photoacoustic imaging

Photoacoustic imaging also uses laser light, but this technique assesses tissue oxygen saturation instead of perfusion. The laser light generates ultrasonic waves that detect the tissue's relative amounts of oxygenated and deoxygenated hemoglobin.^185^ In rats and mice, photoacoustic imaging could detect intestinal ischemia and it correlated well with histology.^186^, ^187^, ^188^ This technique is seen as an emerging imaging modality for specific fields in human medicine, but its limited availability and complicated technique restrict its current use in clinical practice.

### 4.3 Oximetry chip technology

Surface oximetry using a modified Clark electrode^189^ has been used to determine serosal pO_2_ in experimental and naturally occurring large intestinal ischemia in horses.^7^, ^190^ However, nonviable colon was only accurately identified in 53% of the cases,^7^ and research in canine and rabbit ischemia models has also yielded varying accuracies.^19^, ^52^, ^122^, ^191^ Furthermore, the equipment is not readily available, and the technique has several limitations, such as the very localized nature of the measurement and the necessity to puncture the intestine with the electrode.^189^ Nonetheless, a more recent study investigated the use of a small chip that could measure pO_2_ following implantation in rat intestine.^192^ This technique enables continuous perioperative pO_2_ measurement, which could be useful for postoperative monitoring of segments with questionable viability. Currently, this chip is not commercially available, and it has not been validated for clinical application.

### 4.4 Optical coherence tomography

Optical coherence tomography is a relatively new imaging modality that can assess tissue structure in real‐time. It can be regarded as optical histology by detecting changes in tissue structure or microcirculation through differences in light reflection.^193^, ^194^ In horses, this modality has only been used to visualize Descemet's membrane detachments.^195^ In other species, studies have mainly focused on intraluminal imaging of gastric lesions, polyps, and inflammatory bowel disease.^196^ However, optical coherence tomography has also successfully been used for real‐time imaging of tissue injury in experimental rodent ischemia and human patients with AMI.^197^, ^198^

## 5 LIMITATIONS

Evaluating the current evidence for real‐time intraoperative intestinal viability assessment, reveals several limitations. First, most studies were performed under experimental conditions. Using experimental ischemia models to validate intestinal viability assessment should be questioned because ischemia models are not necessarily representative of clinical ischemia. In some studies, the accuracy of a modality was based on the ability to differentiate between normal and ischemic intestines. This does not represent the clinical situation, where ancillary methods are mainly needed for cases with dubious viability according to clinical judgment. Looking at the available clinical trials, this reveals that most were single‐center and that they were not controlled, randomized, or blinded. Most experimental studies that compared different modalities were also not blinded when comparing different modalities. For the validation of a device for intraoperative use, blinding the surgeon for the result of the measurement can ensure that intraoperative decisions are taken irrespective of the measurement. Not doing so poses a risk of bias, which needs to be considered when interpreting study results. Only a few authors have reported cutoff values or reference ranges, and many studies had low case numbers or a heterogeneous case population. Furthermore, research groups tended to focus on one single modality, and initial positive results could not always be matched in subsequent trials.

## 6 FUTURE DIRECTIONS

The limited accuracy of clinical intestinal viability assessment has long been established, and the intraoperative use of ancillary diagnostics may improve these assessments. The considerable analogies between intestinal diseases in different species can be used to adapt existing strategies for use in horses. When extrapolating the research destined for companion animals and humans to the equine intestine, one should critically reflect on which ischemia model and outcome variable was used. Regardless of interspecies differences, these studies give valuable information on the functionality of a diagnostic modality in intestinal tissue, as well as the quantitative potential and possible limitations. Modalities such as tissue oximetry, ICG FA, and thermography are available in veterinary practice, and larger facilities may already be in possession of appropriate devices, such as NIRS monitoring equipment. Although these devices are currently not used for intestinal viability assessment in horses, they can potentially be used during colic surgery. However, the available evidence also highlights limitations that must be investigated in equine‐specific strangulating lesions before basing any intraoperative decisions on these measurements. The techniques discussed are minimally invasive, potentially justifying research in clinical patients following initial testing of the modality. Consequently, validating techniques for an equine colic surgery setting may be facilitated by collecting data from multiple centers and higher case numbers. Furthermore, blinded clinical trials are required in both veterinary and human medicine to determine the accuracy and cutoff values of the different techniques. Interspecies exchange may facilitate the adaptation of quantitative assessment strategies and establishing reference ranges for intestinal saturation or perfusion indices.

## AUTHOR CONTRIBUTIONS

Verhaar N, DVM, PhD, DECVS: Contributed to conception and design, data acquisition, analysis and interpretation and preparation of manuscript and figures. Geburek F, Dr med vet, DECVS, DECVSMR: Contributed to conception and design and edited the draft. All authors provided a critical review of the manuscript and endorse the final version. All authors are aware of their respective contributions and have confidence in the integrity of all contributions.

## CONFLICT OF INTEREST STATEMENT

The authors have no conflict of interest to declare.
